# Molecular detection of toxoplasmosis in wild rats using loop-mediated isothermal amplification assay

**DOI:** 10.14202/vetworld.2024.1575-1580

**Published:** 2024-07-24

**Authors:** Heni Puspitasari, Lucia Tri Suwanti, Mufasirin Mufasirin, Kusnoto Kusnoto, Ira Sari Yudaniayanti, Boedi Setiawan, Endang Suprihati, Eduardus Bimo Aksono, Dwi Priyo Widodo, April Hari Wardhana, Makoto Matsubayashi, Elly Nur Indasari

**Affiliations:** 1 Doctoral Program of Veterinary Science, Faculty of Veterinary Medicine, Universitas Airlangga, Surabaya 60115, Indonesia; 2 Toxoplasma Study Group, Institute of Tropical Disease, Universitas Airlangga, Surabaya 60115, Indonesia; 3 Division of Parasitology, Faculty of Veterinary Medicine, Universitas Airlangga, Surabaya 60115, Indonesia; 4 Division of Clinic Veterinary, Faculty of Veterinary Medicine, Universitas Airlangga, Surabaya 60115, Indonesia; 5 Division of Veterinary Medicine, Faculty of Veterinary Medicine, Universitas Airlangga, Surabaya 60115, Indonesia; 6 Department of Parasitology, Faculty of Veterinary Medicine, Universitas Gadjah Mada, Yogyakarta 55281, Indonesia; 7 Research Center for Veterinary Sciences, Organization for Health, National Research Center for Veterinary Sciences, Cibinong, 16911, Indonesia; 8 Department of Veterinary Immunology, Graduate School of Veterinary Sciences, Osaka Metropolitan Univesity, Osaka, Japan, 598-8531; 9 Department of Veterinary Public Health, Faculty of Veterinary Medicine, Universitas Brawijaya, Malang, 65144, Indonesia

**Keywords:** loop-mediated isothermal amplification, public health, tissue cyst, toxoplasmosis, wild rats

## Abstract

**Background and Aim:**

Toxoplasmosis is caused by the parasite *Toxoplasma gondii*. Cats are the only known hosts that excrete resistant oocysts. Wild rats serve as crucial reservoirs and intermediate hosts for *T. gondii’s* survival and dissemination. Consuming soil and water containing oocysts can lead to illness. This study aimed to estimate the prevalence of toxoplasmosis in wild rats through molecular detection as an indicator of environmental contamination in Surabaya.

**Materials and Methods:**

One hundred rats were collected from the three areas (housing, dense settlements, and traditional markets) and distributed into the five zones: West, East, Central, North, and South of Surabaya. Brain tissue samples were extracted using a Geneaid™ (New Taipei City, Taiwan) DNA isolation kit and analyzed through the loop-mediated isothermal amplification (LAMP) method.

**Results:**

The study analyzed brain tissue from 100 wild rats, consisting of 77 *Rattus tanezumi* and 33 *Rattus norvegicus*, displaying 30% LAMP positivity. The study revealed that 30% (30/100) of wild rats tested were infected with *T. gondii*. The molecular prevalence rate in male rats was 32.35% (22/68), compared to females with 25% (8/32). 41.9% of the housing population, 33.3% of traditional markets, and 22.6% of dense settlements had the highest molecular prevalence. The high positive molecular rate at the trapping site can be attributed to cats and dense populations.

**Conclusion:**

Thirty percentage wild rats were tested positive for toxoplasmosis in Surabaya, East Java, Indonesia using LAMP method. Implementing strict control and monitoring is crucial in preventing the transmission of diseases from wild rats to humans. It is necessary to carry out further research related to genetic analysis of *T. gondii* to determine the type of *T. gondii* that infects animals and humans in Surabaya through bioassay and molecular test.

## Introduction

Immunocompromised and pregnant women are susceptible to experiencing severe health problems from *Toxoplasma gondii* infections, which are generally asymptomatic in healthy individuals [1]. Cats are the only known hosts for excreting-resistant oocysts. Humans, other mammals, and birds can act as intermediate hosts. Although *T. gondii* infections are typically asymptomatic in healthy people, they can cause major health issues in immunocompromised and pregnant women [1]. *T. gondii*, a common intracellular parasite, causes toxoplasmosis in humans and animals. As important reservoirs and intermediate hosts, rodents significantly contribute to the spread and upkeep of *T. gondii*. The parasite can survive as cysts within an animal’s body and serve as a potential source of infection for predators and their offspring [2]. From a medical, veterinary, and economic perspective, toxoplasmosis-related illness holds great significance [3].

*T. gondii*, a parasite in the Phylum *Apicomplexa*, is a globally distributed zoonosis [4]. Acute toxoplasmosis outbreaks in humans are predominantly caused by oocysts [5]. Though complex, *T. gondii’s* life cycle does not fully explain how the parasite moves from definitive to intermediate hosts. In the “classical” complex life cycle, felids, or domestic and wild cats, act as definitive hosts (DHs) while their prey acts as an intermediate host [6]. Cats consider mice as prey and intermediate hosts due to rodents being their usual prey [7]. *T. gondii’s* life cycle may end with oocysts providing access to contaminated meat for cats, thereby propagating the infection to additional hosts. Although it is notable in mice, *T. gondii* is infrequent based on various serological investigations [8]. In polymerase chain reaction (PCR)-based studies of *Mus domesticus* and other rat species, a significant presence of *T. gondii* has been documented [9].

Loop-mediated isothermal amplification (LAMP) method demonstrates unique user-friendliness, high efficiency, sensitivity, simplicity, and specificity when applied in isothermal conditions, according to Fallahi *et al*. [10] and Mahmoudvand *et al*. [11]. This technique was developed by Mirahmadi *et al*. [12] in 2000, has been effectively used to detect parasitic infections such as *T. gondii*, *Schistosoma* spp., *Fasciola* spp., *Babesia* spp., *Trypanosoma* spp., *Cryptosporidium* spp., *Entamoeba histolytica*, and *Plasmodium* spp. LAMP was created in 2000 to improve the sensitivity and specificity of nucleic acid amplification [13]. Currently, this method is used to diagnose various infectious diseases [14]. LAMP amplification was used in place of PCR, which not only removed the need for an advanced heat cycler but also greatly reduced the amount of time needed for amplification due to its DNA amplification efficiency, which was greater than exponential [13]. The LAMP-based assays were exceptionally sensitive and specific. The need for expensive thermocyclers and lengthy adjustments to technical cycling conditions has been obviated by LAMP’s self-cycling strand displacement activity [15]. Liang *et al*. [16], Rivera and Ong [17], and Singh *et al*. [18] confirmed the effectiveness of their LAMP-based assays for disease diagnosis. LAMP amplification efficiency is shown to be higher than that of PCR in previous research [19].

Wild rats play a significant role in the spread of toxoplasmosis. A rat’s altered behavior from parasite infection facilitates cats preying on them, thus completing the parasite’s life cycle. Felids typically acquire the infection from consuming wild rodents, birds, or oocysts shed by other cats. Over the past 10 years, extensive research has been conducted on the worldwide distribution of rodent toxoplasmosis. The prevalence of a diagnosis is influenced by the geographic region, type of diagnosis, and animal type. Iran has reported a high frequency of 86% using PCR [20]. The overall prevalence of toxoplasmosis is significantly influenced by population density, dietary habits, hygiene, and the prevalence of both interspecies hosts and DHs. In Africa, reports of naturally occurring murine toxoplasmosis are scarce. *Mus musculus*, *Rattus rattus*, and *Rattus norvegicus* were the rodent species for which toxoplasmosis in Africa was known [21]. This study aimed to estimate the prevalence of toxoplasmosis in wild rats through molecular detection with LAMP assay specifically the surface antigen 1 (SAG-1) as an indicator of *T. gondii* contamination in the environment in Surabaya, East Java, Indonesia.

## Materials and Methods

### Ethical approval

The study was approved by the Animal Care and Use Committee of the Faculty of Veterinary Medicine at Airlangga University (Approval number 2.KEH.080.07.2022).

### Study period and location

The study was conducted from May to July 2023 in Surabaya. Wild rats were trapped in housing, dense settlements, and traditional markets. Surabaya has five regions: East, West, South, North, and Central, where wild rats were captured using mouse traps [22]. The samples were processed at Veterinary Parasitology Laboratory, Faculty of Veterinary Medicine, Universitas Airlangga for parasitological test. Molecular LAMP assay was performed at Veterinary Parasitology Laboratory, Faculty of Veterinary Medicine, Universitas Gadjah Mada and Institute of Tropical Disease, Universitas Airlangga.

### Sampling technique

For this cross-sectional study, we collected wild rats from the field (housing, dense settlements, and traditional markets).

### Trapping rats

A live trap was used to catch rats. Five Surabaya areas (North, West, South, East, and Central Surabaya) had night traps installed; each area’s sampling locations included housing, dense settlements, and the traditional markets. The number of traps set up at each location for 2 months, used a sample of about 100 wild rats. No anticoagulants were used during the blood collection, brain testing for parasitological analyses, or LAMP assay [22].

### LAMP assay

A total of 25 μL of the reagent was used in the LAMP reaction; this included 1 μL MgSO_4_ (150 mM), 3.5 μL deoxynucleoside triphosphate (dNTP) mix (10 mM), 2.5 μL BST thermopol buffer (10× reaction) (New England Biolabs), 1 μL forward inner primer (50 μM), 1 μL backward inner primer (50 μM), 1 μL F3 primer (5 μM), and 1 μL B3 primer (5 μM); 1 μL loop forward primer (25 μM), 1 μL loop backward primer (25 μM), 2 μL template DNA, 1 μL Bst Polymerase (8000 U/mL), and 9 μL distilled water [13]. Using PrimerExplorer V4 software (http://primerexplorer.jp/lampv5e/ index.html), the LAMP primer was created based on the SAG-1 gene of the local isolate *T. gondii*, as per GenBank Accession No. AY651825. The primers used in this study are listed in Table-1.

**Table-1:** LAMP primers based on the SAG 1 gene of the local isolate *Toxoplasma gondii* designed with PrimerExplorer V4.

Primer LAMP	Sequence (5’ --> 3’)
F3 (Forward outer primer)	GCTCCTTGATTCCTGAAGCA
B3 (Backward outer primer)	GCTGTCTGCACCGTAGGA
FIP (F1c-F2) (Forward internal primer)	GCGTTGTCACGGGGAACTTCT-TTTT-GGACGGGGGATTCTGCTA
BIP (B1c-B2) (Backward internal primer)	CAAGGGAGACGACGAACAGAG-TTTT-GCGACATTATTGACGACCGA
LF (Loop forward)	TTTGATGCCTGCCGTGTC
LB (Loop backward)	TCACAGTGACAGTACAAGCC

The LAMP reaction was conducted for 45 min at 63°C and then stopped for 20 min at 80°C with Tm values for optimum efficiency and specificity as described by Notomi *et al*. [13]. A thermocycler or a water bath can be used in the LAMP DNA amplification process with the same outcomes. Once the amplification procedure was completed, 1 µL of 1000× concentration SYBR green I (Lonza Bioscience, Switzerland) was added to read the findings. A negative LAMP test will remain orange, whereas a positive LAMP result will turn yellow–green. Positive findings glow brightly in a greenish–yellow hue, and ultraviolet (UV) light can also be used to observe the differences in the results. In this study, we used positive and negative control to detect contamination reactions. For negative control, we used ddH20 as a template. For positive control, we used DNA template from *T. gondii* isolate RH strain. To identify the DNA bands that formed, the LAMP product findings were followed by electrophoresis on a 1.5% agarose gel [13]. The results revealed typical bands of varied widths.

## Results and Discussion

### Prevalence of toxoplasmosis among wild rats

The findings of *Toxoplasma*’s analysis of gondii cysts in 100 wild rats that were gathered in Surabaya from May to July 2023.

Molecular detection of brain tissue for the presence of bradyzoite provides higher results in the LAMP assay, which was 30% (30/100). Positive LAMP results indicate the prevalence of toxoplasmosis in wild-caught mice with the gene encoding SAG1. The results of positive LAMP analysis were not significantly different (p < 0.05) between genders, female rats was 32.35% and male rats was 25%. In the subdistricts, positive LAMP results in each district were also not significantly different from the highest values, namely central and southern Surabaya at 50%, north at 28.27%, and east at 25.58%. The habitat shows a high positive LAMP value in dense settlements (41.9%), followed by traditional markets (33.3%), and housing (22.6%). The *R. tanezum*i and *R. norvegicus* have the same LAMP positivity rate (Table-2).

**Table-2:** Molecular detection in brain tissue for the presence of bradyzoites in the LAMP test.

Category	Variables	Prevalence LAMP (%)
Gender	Male	32.35 (22/68)
	Female	25 (8/32)
Subdistrict	West Surabaya	0 (0/16)
	Central Surabaya	50 (13/26)
	South Surabaya	50 (4/8)
	East Surabaya	25.58 (11/43)
	North Surabaya	28.57 (2/7)
Habitats	Housing	41.9 (13/36)
	Dense settlements	22.6 (7/31)
	Traditional markets	33.3 (11/33)
Species	*Rattus tenezumi*	27.27 (21/77)
	*Ratttus norvegicus*	27.27 (9/33)

LAMP=Loop-mediated isothermal amplification

The prevalence of toxoplasmosis in children in some of the world’s most populous regions is as follows: 24.24% in Africa, 18.07% in Asia, 4.63% in Australia, 12.58% in Europe, 8.21% in the United States, and 19.40% in the South [22]. As the primary source of infection for cats and their relatives as well as reservoirs and carriers of the disease, rodents are crucial to the maintenance of the life cycle and epidemiology of *T. gondii* [23]. The epidemiology of *T. gondii* infection is influenced by several variables, including the density of cats and intermediate hosts such as rodents, temperature and humidity levels in the surrounding environment, geographic location, and food processing [24, 25].

The seroprevalence of toxoplasmosis in wild rats in Surabaya has been reported by Puspitasari *et al*. [22] at 31% immunoglobulin (Ig) G (30%) and IgM (1%) and confirmed by microscopic examination for the presence of bradyzoite cyst stages in the brain in 19% of the 100 wild rats. Studies have been conducted to determine the prevalence of *T. gondii* in wild rat populations around the world. The rate of infection with *R. rattus* was 24.41% (31/127) in Iran using the immunochromatographic assay [26]. The rate of infection with *R. norvegicus* was 0.8% (2/238) in Grenada, West Indies, using the modified agglutination test (MAT) [27]. The rate of infection with *R. norvegicus* and *Rattus* *flavipectus*was 3.2% (7/217) in China with the MAT [28]. The rate of infection with *R. norvegicus* was 60% (50/83) and with *R. rattus mindanensis* was 50% (37/74) in the Philippines using the TOXOCELL Direct AD Agglutinations Kit [29]. The rate of infection with *R. norvegicus* was 35% (82/235) in the United Kingdom [30]. The rate of infection with *M. musculus* was 0.3% (2/588) and with *R. norvegicus* was 0% in Iowa, the USA [31]. for the rate of infection with *R. rattus* was 56% (56/100) in Iran with the MAT [25].

Based on a systematic review and meta-analysis [2], the seroprevalence of *T. gondii* in rodents is based on geographical regions, i.e., in Africa, with nine studies showing seroprevalence as much as 24% (0%–48%) with a total positive sample of 311/3058. Australia, with two studies, showed seroprevalence as high as 4% (3%–6%), with total positive samples of 53/837. Europe, with 21 studies, shows seroprevalence as low as 1%, with total positive samples of 454/5991. Asia, with 26 studies, shows seroprevalence as low as 4% (3%–5%), with an overall positive sampling of 650/6239. North America, with 28 studies, shows 5% seroprevalence (4%–7%), with all positive samplings of 433/7942. South America, with 20 studies, shows a total seroprevalence of 18% (14%–23%) with a total positive sample of 362/2.154 [2].

Based on a comprehensive review Dubey *et al*. [20], global prevalence of rodent toxoplasmosis over the past 10 years was conducted. Animal species, geographic region, and diagnostic techniques all affect prevalence. Using the PCR, a high prevalence of 86% has been recorded in Iran [20]. The most prevalent wild rat in Nigeria, *Zyzomys pedunculatus*, had a prevalence of 76.2% (64/84) according to a molecular PCR test of *T. gondii* with the B1 gene [32]. The prevalence of toxoplasmosis is influenced by various factors, such as population size, feeding patterns, societal hygiene, and the abundance of definitive and intermediate hosts. Few naturally occurring murine toxoplasmosis reports exist in Africa [21].

### Molecular detection of the LAMP SAG-1 gene

**Figure-1:**
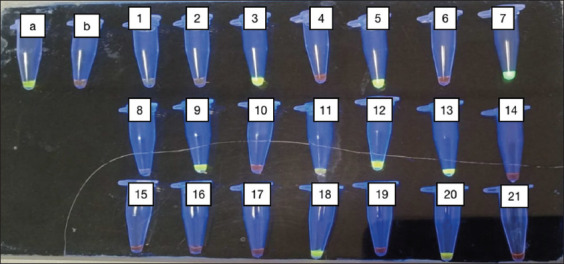
Loop-mediated isothermal amplification products from SAG-1 gene *Toxoplasma gondii* parasite. Using SYBR Green I dye (green color shows positive reaction). (a) = Positive control, (b) = Negative control, Positive results = 3, 5, 7, 9, 11, 12, 13, 18, 20, and Negative results = 1, 2, 4, 6, 8, 10, 14, 15, 16, 17, 19, 21.

**Figure-2:**
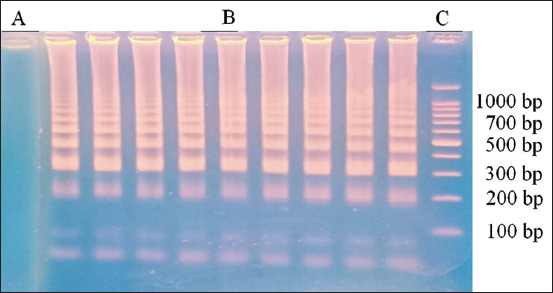
Agarose gel analysis of LAMP assay for the specific detection of *Toxoplasma gondii* DNA based on SAG-1 gene amplification. (A) Negative control, (B) Positive control (C) 100 bp molecular weight marker. LAMP= Loop-mediated isothermal amplification

The LAMP method uses color changes to detect the presence of possible toxoplasmosis in brain samples from wild rats. A microtube turning green indicated a positive result for toxoplasmosis, whereas turning orange showed a negative result using SYBR Green I dye and UV light at 365 nm (Figure-1). The positive result continued with electrophoresis agarose gel for the specific detection of *T. gondii* DNA based on SAG-1 gene amplification. Lane 1: 100-bp molecular weight marker (Figure-2).

Fallahi *et al*. [35] have investigated diverse molecular methods for diagnosing *T. gondii* using distinct targets and techniques. We identified *T. gondii* using a LAMP method specifically targeting the B1 gene [33], which delivers a very high sensitivity. The genomic target of B1 was selected for the current study because molecular markers that target genes such as B1 and conserve the repetitive area of the *T. gondii* genome have good sensitivity and specificity for isolated clinical samples [10]. The LAMP method accurately identified *T. gondii*, consistent with another research [12].

The 4–6 primers used in the present reaction, which target 6–8 distinct internal areas on the B1 gene as the target DNA, increase the diagnostic sensitivity of the LAMP assay [10]. Under isothermal conditions (64°C), LAMP offers the opportunity to amplify DNA quickly with excellent specificity and efficiency, acting as a recombinant DNA technology [13]. The technique employs four separate primers, each binding to the target DNA at six unique sequences. Unlike conventional PCR, this method simplifies the process, reduces the cost, and functions without the need for purified DNA for successful amplification. A single-tube reaction can be completed using an easy incubator, with results obtained in an hour. The rapid amplification, user-friendly design, and straightforward readout methods contribute significantly to the reliability and effectiveness of early infection detection [34]. This molecular identification method shows great potential in underdeveloped countries with high *Toxoplasma* prevalence. The LAMP method relies on the genes SAG-1, SAG-2, and B1 from *T. gondii* [35, 36].

## Conclusion

Based on research results showing the high prevalence of molecular toxoplasmosis in wild rats in Surabaya, displaying 30% LAMP positivity, it is necessary to carry out further research related to genetic analysis of *T. gondii* to determine the type of *T. gondii* that infects animals and humans in Surabaya through bioassay and molecular tests.

## Authors’ Contributions

LTS: Concept and designed the study, coordinated the study, and drafted the manuscript. HP: Performed LAMP and a literature review. ISY, KK, ENI, MM, and BS: Parasitological test and sampling analysis. ES, EBA, DPW, AHW, and MaM: Sampling analysis and revised the manuscript. All authors have read, reviewed, and approved the final manuscript.
